# Medizinethische Herausforderungen der Phagentherapie – informierte Einwilligung, Studiendesign und Heilversuch

**DOI:** 10.1007/s00103-025-04056-y

**Published:** 2025-05-06

**Authors:** Hans Zillmann

**Affiliations:** Seminar für Philosophie, Universität Erfurt, Alfred-Weber-Platz 4, 99089 Erfurt, Deutschland

**Keywords:** Medizinethik, Bakteriophagen, Translation, Autonomie, Gerechtigkeit, Medical ethics, Bacteriophages, Translation, Autonomy, Justice

## Abstract

Die Antibiotikakrise steigert das Interesse an Bakteriophagen und damit in Zusammenhang stehenden Therapieansätzen. Eine Translation der Phagentherapie in die klinische Anwendung steht vor verschiedenen ethischen Herausforderungen. Diese betreffen u. a. die informierte Einwilligung des Patienten auf Grundlage einer Patientenaufklärung sowie offene Fragen eines ethisch abgesicherten Studiendesigns. Zudem ergeben sich in Hinsicht auf die Zugangsgerechtigkeit aus der aktuellen Situation der Phagentherapie in Deutschland weitere ethische Probleme. Dieser Übersichtsbeitrag reflektiert die 3 genannten Aspekte der Phagentherapie. Im Ergebnis zeigt sich, dass Phagentherapie keine Anforderungen an die Patientenaufklärung stellt, die nicht durch Anpassungen der Aufklärung gelöst werden könnten. Fragen an ein angepasstes Studiendesign ergeben sich vor dem Hintergrund des Placebodilemmas, möglicher Resistenzen gegen Antibiotika sowie der erwartbar geringen Zahl an Studienteilnehmern. Es kann versucht werden, mit angepassten Studiendesigns auf diese Herausforderungen zu reagieren. Dadurch weichen die Studien vom Goldstandard, den randomisierten, kontrollierten und doppelt verblindeten Studien, ab. Hier muss eine Abwägung zwischen Evidenzanspruch und Schutz der Studienteilnehmer vorgenommen werden. Aus ethischer Sicht ist der Schutz der Teilnehmer stärker zu gewichten. Die aktuelle Situation der Phagentherapie in Deutschland stellt ein ethisches Problem dar, denn sowohl der individuelle Heilversuch als auch ein zu beobachtender Medizintourismus stehen dem Anspruch auf Zugangs- und Verteilungsgerechtigkeit im Weg. Um diesen Status zu überwinden, stellt die Magistralrezeptur einen möglichen Zwischenschritt dar, bis Klarheit über ein angemessenes Studiendesign gewonnen wurde.

## Abstract (en)

The antibiotics crisis is increasing interest in bacteriophages and related therapeutic approaches. The translation of phage therapy into clinical application faces various ethical challenges. These include the patientʼs informed consent based on patient information as well as open questions regarding an ethically sound study design. In addition, further ethical problems arise from the current situation of phage therapy in Germany with regard to equity of access. This article reflects on the three aspects of phage therapy mentioned above. The results show that phage therapy does not pose any requirements for patient information that could not be solved by adapting the information provided. Questions regarding an adapted study design arise against the background of the placebo dilemma, possible resistance to antibiotics and the expected small number of study participants. An attempt can be made to respond to these challenges by adapting the study design. As a result, the studies deviate from the gold standard, which is randomized, controlled and double-blind studies. A balance must be struck here between evidence requirements and the protection of study participants. From an ethical point of view, the protection of participants must be given greater weight. The current situation of phage therapy in Germany poses an ethical problem, as both the individualised therapeutic trial and the resulting medical tourism that can be observed stand in the way of the demand for fair access and distribution. In order to overcome this status, the magistral prescription represents a possible interim step until clarity has been gained about an appropriate study design.

## Einleitung

Die Notwendigkeit einer Alternative für Antibiotika wird zunehmend deutlich [1]. Jährlich sterben ca. 5 Mio. Menschen weltweit an bakteriellen Infektionen mit antibiotikaresistenten Erregern und bis 2050 wird ein Zuwachs auf bis zu 10 Mio. erwartet.^1^ Daraus ergibt sich ein globales Versorgungsproblem mit wirksamen Therapien gegen bakterielle Erkrankungen. Dies steigert das Interesse, Bakteriophagen zur Therapie einzusetzen [2]. Bei einer Translation der Phagentherapie in die klinische Anwendung ergeben sich ethische Herausforderungen.

Dieser Übersichtsbeitrag beschäftigt sich mit 3 Bereichen der Phagentherapie, denen ethische Herausforderungen innewohnen. Einerseits betrifft dies Herausforderungen, vor denen eine Translation der Phagentherapie in Deutschland in die klinische Anwendung steht. Andererseits handelt es sich um ethische Erwägungen zum Status quo der Phagentherapie in Deutschland. Erstens werden Fragen der informierten Einwilligung diskutiert, denn Phagentherapie ist für Patienten ein weitestgehend unbekanntes Feld mit möglicherweise besonderen Herausforderungen im Prozess der Patientenaufklärung. Zweitens stellen sich in Zusammenhang mit Phagentherapie Fragen nach einem möglichen Studiendesign. Das weitestgehende Fehlen klinischer Studien kann als eine zentrale ethische und regulatorische Herausforderung für die Phagentherapie in Deutschland angesehen werden [3]. Drittens sind Fragen der Gerechtigkeit und des Zugangs zentral.

Andere ethische Fragen, die sich in Zusammenhang mit Phagentherapie ergeben können, wie z. B. zu Missbrauchspotenzialen sowie bei einer möglichen Kommerzialisierung, werden in diesem Beitrag nicht gestellt. Die hier vorgenommenen Reflexionen beziehen sich auf aktuelle praktische ethische Herausforderungen einer Translation der Phagentherapie in die klinische Anwendung. Dieser Beitrag versteht sich als Diskussionsaufruf zu weiterer ethischer Forschung zu Bakteriophagen und Phagentherapie.

## Medizinethische Grundlegung

Neben anderen Theorien und Methoden, wie z. B. Ansätzen deontologischer oder konsequenzialistischer Ethiken, ist die moderne Medizinethik durch 4 Prinzipien geprägt [4], die dabei helfen sollen, die ethische Tragfähigkeit medizinischen Handelns zu bewerten und sicherzustellen. An dieser Stelle soll der Beitrag für eine erste medizinethische Orientierung sorgen. Die folgenden Darstellungen werden daher auf die 4 Prinzipien der Medizinethik eingegrenzt. Diese Prinzipien könnten anders begründet und inhaltlich ausgearbeitet werden, als es hier geschieht [6]. Auch hier sei der Aufruf zu weiterer ethischer Forschung zur Begründung und inhaltlichen Ausarbeitung medizinethischer Erwägungen zur Phagentherapie vorgebracht.

Das *Prinzip der Autonomie* ist im Kontext ärztlichen oder medizinischen Handelns dann gewahrt, wenn dem Patienten notwendige Voraussetzungen für eine informierte und freiwillige Zustimmung zum geplanten Vorgehen gegeben sind [7]. Hier sind nicht nur Fragen der Patientenaufklärung relevant, aber sie sind zentral (siehe Abschnitt zu Fragen der Einwilligung). Vonseiten der Ärzte wird Autonomie dann zugeschrieben, wenn der Patient bestimmte Fähigkeiten hat und über ein bestimmtes Wissen verfügt. Für die Wissensvermittlung ist der Arzt verantwortlich.

*Das Prinzip des Nichtschadens* soll sicherstellen, dass medizinisches Handeln keinen unnötigen bzw. unverhältnismäßigen Schaden für den Patienten zur Folge hat [4]. Daraus leitet sich auch der Anspruch ab, Patienten nur diejenigen Stoffe zu verabreichen, bei denen evidenzbasiert davon ausgegangen werden kann, dass sie ihnen nicht schaden bzw. dass ihr Nutzen in einem vertretbaren Verhältnis zum möglichen Schaden steht. Die Einhaltung dieses Prinzips erfordert eine ausreichende Datenlage, auf deren Grundlage potenzielle Schäden eingeschätzt werden können. Die Phagentherapie steht diesbezüglich vor besonderen Herausforderungen, denn bisher existieren neben Einzelfallberichten und Übersichtsarbeiten nur wenig Anhaltspunkte für eine solche Bewertung. Weitere medizinische Forschung ist auch aus ethischer Sicht gefordert.

Gemäß dem *Prinzip des Wohltuns* soll ärztliches Handeln grundsätzlich der Gesundheit und dem Leben der Patienten dienen. Dieses Prinzip kann in bestimmten Situationen voraussetzungsreich sein oder mit anderen Prinzipien, wie dem der Autonomie, in Konflikt geraten, wenn sich der Patient z. B. gegen eine aus Sicht des Arztes Erfolg versprechende Therapie ausspricht [4]. Aus dem Prinzip des Wohltuns kann die Forderung an den Arzt abgeleitet werden, seinem Patienten die bestmögliche der verfügbaren Behandlungen zukommen zu lassen. Die Frage nach der besten Eignung kann letztlich nur vor dem Hintergrund wissenschaftlicher Evidenz beantwortet werden. Dies stellt in Zusammenhang mit Bakteriophagen eine Herausforderung dar (siehe Abschnitt zum Studiendesign).

Aus dem zweiten und dritten Prinzip der Medizinethik entspringt die Norm, dass Wirkstoffe, die Menschen verabreicht werden, erst auf ihre Wirksamkeit und vor allem Sicherheit getestet werden müssen. Eine ethische Reflexion kann keine Aussagen darüber treffen, inwieweit eine evidenzbasierte Risiko-Nutzen-Kalkulation für die Phagentherapie bereits möglich ist. Gleichzeitig kann jedoch aus ethischer Perspektive festgehalten werden, dass Wege gefunden werden müssen, diese Frage mittelfristig zu beantworten. Denn mit Bakteriophagen scheint ein möglicher Wirkstoff gegen bakterielle Erkrankungen und eine Alternative zu Antibiotika verfügbar zu sein. Besonders angesichts der Antibiotikakrise könnte dies von Bedeutung für Leben und Gesundheit sehr vieler Menschen sein. Eine geregelte Verfügbarkeit von Bakteriophagen in Deutschland kann nur durch eine evidenzbasierte Risiko-Nutzen-Kalkulation anvisiert werden.

Das 4. medizinethische *Prinzip Gerechtigkeit* soll individuelle und soziale Gerechtigkeit im Gesundheitswesen garantieren. Es soll sicherstellen, dass Verfügbarkeit und Zugang zu bestimmten medizinischen Maßnahmen gleich verteilt sind und ein gemeinschaftlich gerechter Weg zur Verteilung von Gesundheitsleistungen gefunden wird [4]. Ein gerechter Zugang und eine gerechte Verteilung können auch vom Grundsatz der Gleichheit abweichen, wenn kompensatorische Gerechtigkeit für benachteiligte Gruppen entstehen soll. Dabei geht es auch um die individuellen und gemeinschaftlichen finanziellen Kosten für die Gesundheitsversorgung [4]. Der Status quo der Phagentherapie in Deutschland scheint diesbezüglich herausfordernd. Zugang haben Patienten nur über einen „individuellen Heilversuch“ oder eine Reise ins Ausland (siehe Abschnitt zu Zugangsproblemen).

## Fragen der Einwilligung

Durch die Patientenaufklärung geben Ärzte den Patienten alle notwendigen Informationen, sodass diese ihre Entscheidung auf Basis eines umfassenden Verständnisses treffen können [4]. Auf diesem Weg wird eine informierte Einwilligung erst möglich, das Recht der Patienten auf Selbstbestimmung geschützt sowie gewährleistet, dass Entscheidungen frei von Zwang und in Kenntnis aller relevanten Informationen getroffen werden können [8].

Beauchamp und Childress haben in ihrem Grundlagenwerk als notwendige Voraussetzungen für Autonomie *Autonomiekompetenz, Freiwilligkeit* und *Verstehen* gesetzt: Autonomiekompetenz zeichnet sich durch die Fähigkeit aus, Informationen verstehen und verarbeiten sowie aus den gegebenen Informationen entsprechende Konsequenzen des eigenen Handelns ableiten zu können [4]. Autonomiekompetenz ist im Wesentlichen etwas, das der Patient mitbringt bzw. der Arzt ihm zuschreibt. Freiwilligkeit gehört zu den Grundprinzipien medizinischen Handelns und sei hier vorausgesetzt. Die dargebotenen Informationen, deren Aufbereitung und Vermittlung liegen in der Verantwortung des Arztes und sollen das Verstehen sicherstellen. Wenn die Umstände ein Gespräch zwischen Arzt und Patient erlauben, stellt sich also die Frage, welche „epistemischen Voraussetzungen“ [9] die autonome Zustimmung eines Patienten zu einer Phagentherapie hat.

Für eine Translation der Phagentherapie in die medizinische Anwendung ist zu klären, ob sich besondere Anforderungen an die Patientenaufklärung stellen, damit eine informierte Einwilligung gewährleistet ist. Nun ist die Phagentherapie nicht nur für Ärzte ein Feld mit Unbekannten. Es kann davon ausgegangen werden, dass Patienten nicht per se über diesbezügliches Wissen verfügen. Das Fehlen umfangreicher klinischer Daten, die Neuheit der Therapie und die individuelle Natur der Behandlung machen es schwieriger, Patienten über Nutzen und Risiken aufzuklären.

Im Folgenden sollen einige mögliche Herausforderungen für die informierte Einwilligung in Zusammenhang mit Phagentherapie diskutiert werden. Diese Aufstellung erhebt keinen Anspruch auf Vollständigkeit und versteht sich als Einladung für weitere Beiträge zum Thema.

Die begrenzte Datenlage und damit einhergehende Unsicherheiten über die Wirksamkeit und Sicherheit des Phagenpräparats müssen Gegenstand der Aufklärung sein und sowohl durch den Patienten als auch den Arzt eingeschätzt werden. Dies stellt im Wesentlichen Anforderungen an das Wissen und Verstehen auf Arzt- und Patientenseite.

Der Hinweis auf fehlende Langzeitstudien ist ein wichtiger und nicht unbekannter Einwand gegen medizinische Neuheiten. Es finden sich in der Literatur dokumentierte Fälle ineffektiver Phagentherapie [10]. Mit dem Hinweis auf Unvollständigkeit listen Suh et al. mögliche Ursachen für Fehlversuche bzw. ineffektive Therapie auf [2]: Fragen der Dosierung, der Verabreichungsfrequenz, der Therapiedauer, der administrativen Wege, der Wechselwirkungen mit Antibiotika, der Wechselwirkung mit anderen Bakteriophagen, das Auftreten von Resistenzen gegen Bakteriophagen sowie eine nicht adäquate, weil vielleicht örtlich zu unspezifische Verabreichung [2].

Gleichzeitig existiert kein vollständiges Unwissen über die Effektivität von Phagentherapie. Erwähnung finden erste Erfolge von Phagentherapie bereits 1919 in einem Pariser Hospital sowie im Zweiten Weltkrieg in der deutschen und alliierten Armee [11]. Das weitestgehende Ende einer Nutzung von Bakteriophagen zur Bekämpfung bakterieller Infektionen in der westlichen Welt wird unter anderem mit der Einführung von Antibiotika, Problemen bei der Herstellung sowie unsachgemäßem Gebrauch der Bakteriophagen begründet [11]. Auch ideologische Konstellationen während der Zeit des Kalten Krieges werden als mögliche Ursachen angeführt [11]. In Georgien wurden Bakteriophagen weiterhin aus der Umwelt isoliert, in einer Phagenbank gesammelt und existieren neben Antibiotika als Therapie für bakterielle Infektionen [12].

Viele Fälle von Phagentherapie werden nicht in Form herkömmlicher Veröffentlichungen in Fachzeitschriften mit Peer Review bekannt gemacht – vielmehr werden sie häufig auf Tagungen vorgestellt oder in Pressemitteilungen veröffentlicht [12]. McCallin et al. veröffentlichten eine Liste von 29 publizierten Fallbeispielen [12]. Auch wenn die Maßstäbe für die Bewertung des klinischen Outcomes variieren, finden sich bei 28 Fallbeispielen Hinweise auf klinische Erfolge, in einem Fall ist der klinische Outcome nicht dokumentiert. Aktuelle dokumentierte klinische Fälle sowie systematische Übersichtsarbeiten machen die Wirksamkeit von Phagentherapie zunehmend deutlich [13, 14]. Bezogen auf 4 randomisierte klinische Studien zur Phagentherapie wird darauf hingewiesen, dass keine „relevante[n] Nebenwirkungen … oder Anzeichen einer therapieassoziierten Toxizität beobachtet wurden“ [11]. Die Häufigkeit von Nebenwirkungen wird auch an anderer Stelle als gering eingestuft [2, 15]. Gleichzeitig wird auf die Notwendigkeit weiterer Forschung hingewiesen [1].

Die evidenzbasierte Medizin soll vor allem die Qualität der Therapie sichern. Sie trägt damit entscheidend zu einer ethischen Absicherung des Vorgehens bei. Die geringe Datenlage und eingeschränkte Evidenz müssen dem Patienten innerhalb einer Patientenaufklärung verständlich gemacht werden, wenn eine informierte Einwilligung zur Phagentherapie hergestellt werden soll.

Auch die Neuheit der Phagentherapie und damit ihre tendenzielle Unbekanntheit könnten der Herstellung einer informierten Einwilligung entgegenstehen, da mit fehlendem oder geringem Vorwissen der Patienten gerechnet werden muss. Vor diesem Hintergrund ist die Neuheit der Phagentherapie vor einer Patientenaufklärung zu beachten und es muss auf eventuelle Hürden für die Patienten Rücksicht genommen werden. Neuheit ist jedoch nichts, das nicht auch andere Therapieansätze bereits mit sich gebracht haben.

Zum Beispiel ist in Deutschland eine genetische Beratung (Gendiagnostikgesetz § 2) vorgeschrieben, wenn der diagnostische Untersuchungszweck auf prädiktive genetische Eigenschaften erweitert wird. Dies soll sicherstellen, dass der Patient die besonderen Aspekte und Herausforderungen einer neuen und komplexen Form medizinischen Handelns, der prädiktiven Gendiagnostik, versteht. Auch innerhalb der Krebstherapie haben Entwicklungen in der individualisierten Medizin zu neuen Anforderungen an die Patientenaufklärung und die informierte Einwilligung geführt [16].

In Zusammenhang mit Phagentherapie stellen sich Fragen, die spezifisch für individualisierte bzw. personalisierte Medizin sind. Die besonderen Herausforderungen einer individualisierten Phagentherapie müssen dem Patienten gegebenenfalls verständlich gemacht werden. Nicht nur aufgrund der aktuellen Regulierungsprobleme in Deutschland, sondern auch aus biologisch-technischen Gründen steht zu erwarten, dass Phagentherapie zwar nicht ausschließlich, aber zu einem gewissen Teil auf Grundlage individuell hergestellter Phagenpräparate oder Phagencocktails durchgeführt wird. Da Bakteriophagen sehr spezifisch sind, müssten andernfalls sehr viele verschiedene Präparate nach guter Herstellungspraxis (GMP) zugelassen werden. Diese Perspektive erscheint wenig erwartbar. Zudem besteht die Herausforderung, dass die bakteriellen Erreger sich verändern und fortwährend nach weiteren Bakteriophagen gesucht werden muss. Auch hier scheint der Weg über GMP wenig realistisch. Phagentherapie führt bezüglich der individuellen Herstellungsweise des Präparats nicht zu neuen Herausforderungen für die Patientenaufklärung, die Umstände der Herstellungsweise müssen allerdings transparent gemacht werden.

Selbst wenn sich Biobanken etablieren sollten, die Bakteriophagen bzw. Phagencocktails für z. B. Krankenhausapotheken zur Verfügung stellen, würden die Hilfsstoffe des Medikaments individuell und bezogen auf Patienten und Infektionsart sowie Infektionsort unterschiedlich sein. Dies könnte aus Patientensicht nicht das Gleiche sein wie die Einwilligung in die Gabe eines standardisiert hergestellten und zugelassenen Medikaments, das man in einer Apotheke erwerben kann. Es könnten z. B. Bedenken der Patienten über die Wirksamkeit des Medikaments bestehen. Auch stellt sich hier die Frage, welches Wissen dem Patienten auf welche Art zugänglich gemacht werden muss, damit davon ausgegangen werden kann, dass er zu einer informierten Einwilligung in der Lage ist. Individuell hergestellte Medikamente und der Umgang damit bezüglich Patientenaufklärung und informierter Einwilligung sind keine Neuheiten.

Notfallanwendungen bringen Herausforderungen für die informierte Einwilligung, denn der Patient könnte risikofreudiger sein, wenn kein zugelassenes Medikament hilft. Dies wirft auch Fragen nach der Autonomie des Patienten auf, denn möglicherweise fällen Menschen andere Entscheidungen, wenn sie mehr Zeit oder Alternativen haben. Es ist zu erwarten, dass dieser Punkt bei Phagentherapie häufig erfüllt sein wird. Und Patienten in Notfallsituationen könnten dazu neigen, die Potenziale zu überschätzen und die Risiken zu unterschätzen [17]. Auch dies kann bei anderen schweren Erkrankungen zutreffen. In derartigen Fällen ist eine ausführliche Patientenaufklärung notwendig. Die Entscheidung, die der Patient dann trifft, muss als Ausdruck seiner Autonomie akzeptiert werden.

Die Patientenaufklärung ist ein bewegliches und anpassungsfähiges Instrument. Besondere Anforderungen an sie können je nach Fall medizinischer, rechtlicher und ethischer Natur sein. Die Herstellung einer informierten Einwilligung durch eine Patientenaufklärung über spezifische Aspekte der Phagentherapie ist unter Anpassung der Aufklärungsinhalte widerspruchsfrei denkbar. In diesem Zusammenhang kann Anschlussforschung empfohlen werden, welche besonderen Aspekte der Phagentherapie in der Patientenaufklärung auf welche Art adressiert werden müssen.

## Ethische Herausforderungen an das Studiendesign

Aus ethischer Sicht sind Studien notwendig, um zu einer genaueren Risiko-Nutzen-Abwägung für Patienten zu gelangen. Eine Herausforderung bei der Forschung zur Phagentherapie ist, dass es an standardisierten Methoden fehlt. Im Gegensatz zu Antibiotika, die in großen *randomisierten kontrollierten Studien *(RCT) getestet werden können, werden Bakteriophagen in kleineren Studien oder sogar Einzelfallberichten untersucht. RCT gelten als die „nachweislich beste Methode, um eine medizinische Fragestellung zu beantworten“ [18]. Es ist strittig, wie viel Evidenz aus Fallstudien abgeleitet werden kann, denn negative Ergebnisse werden seltener veröffentlicht [2]. Den positiven Fällen könnte eine unbekannte Anzahl von negativen Fällen gegenüberstehen.

Aufgrund der großen Spezifizität von Bakteriophagen besteht eine Herausforderung darin, genügend Teilnehmer für eine Studie zu gewinnen. Aktuell ist nicht ersichtlich, wie dies bewältigt werden sollte. Entweder müssten sehr viele verschiedene Studien durchgeführt werden, da immer nur die Sicherheit und Wirksamkeit von bestimmten Bakteriophagen als Therapie für bestimmte bakterielle Infektionen geprüft werden könnte. Diese Studien hätten dann eine kleine Teilnehmerzahl. Oder polyvalente Bakteriophagen bzw. Phagencocktails müssten Gegenstand der klinischen Studien sein, was die Zahl notwendiger Studien senken und die Zahl potenzieller Teilnehmer steigern könnte. Da sich Bakterien verändern, stünde auch die Frage im Raum, wie lange polyvalente Bakteriophagen bzw. Phagencocktails wirksam wären.

Es gibt Möglichkeiten beschleunigter Verfahren, die rechtlich auf EU-Ebene geregelt sind (Richtlinie 2001/83/EG; Verordnung (EG) Nr. 1234/2008; Leitlinien EMA). Hier wird die Änderung zugelassener Arzneimittel sowie deren Bewertung innerhalb von 3 Risikostufen geregelt. Die rechtswissenschaftliche Forschung könnte Klarheit darüber schaffen, inwieweit diese Regulierungen auch im Falle von Phagentherapie angewendet werden können. Dann könnten schnellere Zulassungen neuer Bakteriophagen als Wirkstoff möglich sein und es müssten nicht fortwährend neue Studien mit geringer Teilnehmerzahl durchgeführt werden.

Sofern Studien mit Schwerstkranken durchgeführt werden sollen, die mit antibiotikaresistenten Bakterien infiziert sind, ergibt sich aufgrund des Placebodilemmas eine weitere ethische Herausforderung an das Studiendesign. Die Gabe von Antibiotika an eine Vergleichsgruppe ist aufgrund der in diesem Fall vorliegenden Resistenzen nicht möglich. Eine Placebogabe in Verbindung mit einer doppelten Verblindung könnte wiederum Gesundheit und Leben der Studienteilnehmer gefährden und stellt einen ethisch problematischen Weg dar [19]. Auch dies ist im Grunde keine unbekannte Herausforderung und es werden verschiedene Wege des Umgangs damit diskutiert.

Ein Vorschlag ist das *Open-Label-Design* [19]. Um zu verhindern, dass die Gesundheit oder das Leben der Teilnehmer aufgrund der Placebogabe gefährdet werden, soll auf die Verblindung verzichtet werden. Sowohl das medizinische Personal als auch die Teilnehmer wissen, wer der Placebogruppe angehört bzw. wer nicht. Dies ermöglicht eine genauere Überwachung der Teilnehmer der Placebogruppe bezüglich ihres Gesundheitszustandes. In diesem Zusammenhang könnte die Implementierung eines *Early-Escape*- oder *Rescue-Protokolls* gewinnbringend sein. Denn dann kann ein Teilnehmer, dessen Zustand sich während der Placebogabe verschlechtert, aus der Placebogruppe genommen und in die Gruppe mit der aktiven Behandlung überführt werden. So wird das Risiko gemindert, dass ein Teilnehmer unnötig leidet oder langfristig Schaden nimmt.

Vor allem das Open-Label-Design geht auf Kosten der naturwissenschaftlichen Evidenz und einer evidenzbasierten ethischen Bewertung. Die Verblindung von Studien soll z. B. subjektive Wahrnehmungen des medizinischen Personals und der Studienteilnehmer aus der Evaluation des Studienverlaufs und der Studienergebnisse ausschließen [18]. Denn es könnte sein, dass die Wahrnehmung des medizinischen Personals oder der Studienteilnehmer aufgrund einer gewissen Erwartungshaltung an den Wirkstoff verändert wird. Hier muss auch auf der Grundlage ethischer Erwägungen entschieden werden, ob die Sicherheit der Teilnehmer oder die Evidenz der Studie höher zu bewerten sind. Es steht zu erwarten, dass diese Entscheidung aus ethischer Perspektive zugunsten der Sicherheit der Teilnehmer und damit zugunsten eines Open-Label-Designs ausfallen könnte.

Einen möglichen Mittelweg stellt die *Einfachblindstudie* dar. Hier wird nur aufseiten der Teilnehmer verblindet. Das medizinische Personal kennt die Gruppenzuordnung und kann so eine genauere Überwachung der Placebogruppe vornehmen.

Eine weitere Möglichkeit, das Placebodilemma zu umgehen, ist ein *Add-on-Design*. In diesem Fall erhalten alle Teilnehmer eine Standardtherapie und zusätzlich bekommt eine Gruppe das zu prüfende Medikament und die andere Gruppe ein Placebo. So wird gewährleistet, dass keine Gruppe ohne Behandlung bleibt und gleichzeitig die Wirkung des neuen Medikaments getestet werden kann. Die Studie erfüllt so die Anforderung der Kontrolliertheit. Dabei ist jedoch wichtig, dass die Standardtherapie sicher und etabliert ist. Im Fall von Phagentherapie scheint dies ein durchaus gangbarer Weg zu sein. Als Standardtherapie stünden zugelassene Antibiotika zur Verfügung. Antibiotikaintoleranzen bei Teilnehmern sowie die Resistenz einer zunehmenden Anzahl von Bakterien gegen Antibiotika stehen diesem Vorgehen allerdings entgegen. Eine Kombination von Antibiotikagabe und Phagentherapie kann einen positiven Effekt auf die Therapie haben [20]. In diesem Zusammenhang wird der Vergleich klinischer Studien, in denen den Teilnehmern ausschließlich Antibiotika verabreicht werden, mit Studien, in denen eine Kombination aus Antibiotika- und Phagengabe erfolgt, empfohlen [2]. Prinzipiell ergeben sich Herausforderungen an die Kontrolliertheit einer RCT, wenn die Zusammenstellung einer Kontrollgruppe nicht möglich ist.

Den beschriebenen Herausforderungen könnte dadurch begegnet werden, dass Studien zur Phagentherapie an Personen durchgeführt werden, deren bakterielle Infektionen nicht schwerwiegend sind und bei denen zu erwarten ist, dass ihre Gesundheit durch die Placebogabe nicht übermäßig leidet. Zudem ist nicht absehbar, ob schwerkranke Personen der Teilnahme an einer Studie zustimmen, bei der sie möglicherweise keine aktive Behandlung erhalten.

Es werden verschiedene Wissenslücken innerhalb der Literatur diskutiert, die die Frage nach einem standardisierten Studiendesign aufwerfen. Auch um regulatorische Hürden zu nehmen, ist es von entscheidender Bedeutung, dass standardisierte Studien zur Phagentherapie durchgeführt werden. Es bieten sich Möglichkeiten, das Studiendesign an die biologisch-technischen Eigenheiten und andere Herausforderungen der Phagentherapie anzupassen. Anpassungen des Studiendesigns ermöglichen Innovationsdynamik und stehen gleichzeitig der Forderung nach höchster Evidenz entgegen [21].

Bis die Herausforderungen des Studiendesigns überwunden sind, könnte die *Magistralrezeptur* ein realistischer und ethisch wünschenswerter Weg sein. Ein Apotheker stellt dabei auf ärztliche Verordnung ein Arzneimittel her. Von Apothekerseite wird dabei entweder auf erprobte Vorgaben zurückgegriffen oder es handelt sich um eine *Individualrezeptur* [22]. Im Falle von Phagentherapie ist davon auszugehen, dass es sich vorerst um Individualrezepturen handelt. Der Etablierung erprobter Vorgaben steht jedoch nichts im Wege.

## Zugangsprobleme

Medikamente gelten in Deutschland dann als marktfähig, wenn sie durch das Bundesinstitut für Arzneimittel und Medizinprodukte, das Paul-Ehrlich-Institut oder die Europäische Arzneimittel-Agentur zugelassen wurden [23]. Dennoch ist es in Deutschland möglich, nicht zugelassene Arzneimittel einzusetzen (individueller Heilversuch), Arzneimittel einzusetzen, die für eine andere Therapie zugelassen wurden (Off-Label-Use), oder Medikamente einzusetzen, deren Zulassung beantragt ist (Compassionate Use). Da es sich bei Phagentherapie um eine nicht zugelassene Therapie handelt, deren Zulassung bisher auch nicht beantragt ist, stellt sie einen *individuellen Heilversuch* dar. Es besteht keine Genehmigungs- oder Anzeigepflicht, lediglich die Pflichten zu Aufklärung und Dokumentation bleiben bestehen [23]. Der individuelle Heilversuch steht Arzt und Patient vor dem Hintergrund einer Risiko-Nutzen-Abwägung und ärztlichen Behandlungsentscheidung prinzipiell offen und kommt z. B. in der Onkologie häufig vor.

In Zusammenhang mit individuellen Heilversuchen ergibt sich die Frage nach der Kostenübernahme durch die Gesetzliche Krankenversicherung (GKV). Eine prinzipielle Verweigerung der Kostenübernahme durch die GKV verstößt gegen die im Grundgesetz geregelte allgemeine Handlungsfreiheit, gegen das Sozialstaatsprinzips sowie das Grundrecht auf Leben und körperliche Unversehrtheit [23]. Neue Wege der verlässlichen Kostenerstattung sind ethisch gefordert, wenn Zugangsgerechtigkeit hergestellt werden soll. Weitere Zugangsprobleme ergeben sich, da keine Kriterien vorhanden sind, nach denen Patienten für einen individuellen Heilversuch ausgesucht werden [17]. Auch hier könnten sozioökonomische Faktoren Einfluss auf den Zugang haben.

Zudem fordert die Einwilligung des Patienten in eine experimentelle Therapie die Patientenaufklärung in besonderer Weise heraus, denn sowohl Erfolgsaussichten als auch Risiken können nur unzureichend durch den Arzt dargestellt werden. Individuelle Heilversuche an nicht einwilligungsfähigen Patienten verlagern dieses Problem auf die gesetzlichen Vertreter [24].

Aktuell befinden sich Patienten in Deutschland in einer unsicheren Situation. Medial berichtet wird über Patienten, die auf eigene Kosten nach Georgien reisen, um sich dort einer Phagentherapie zu unterziehen. Die Gerechtigkeits- und Zugangsprobleme, die sich aus dieser Situation ergeben, liegen offen zutage: Es sind nur bestimmte Personen, denen die Möglichkeit offensteht, in ein anderes Land zu reisen. Diese Personen müssen über ein bestimmtes Wissen verfügen, sie müssen physisch oder durch Unterstützung die Möglichkeit haben, eine Reise anzutreten. Und sie müssen über die finanziellen Mittel für eine solche Reise verfügen. Daraus ergibt sich ein ungleicher Zugang zu dieser Möglichkeit. Dies fordert das Prinzip der Gerechtigkeit heraus.

Ethisch erscheint es daher kaum akzeptabel, dass es langfristig bei dieser Situation bleibt. Die regulatorischen Herausforderungen werden bereits diskutiert und Lösungsvorschläge erarbeitet, um eine Zulassung zu ermöglichen [3, 25]. Auch die Frage der Kostenübernahme durch die GKV muss aus ethischer Sicht beantwortet werden. Der individuelle Heilversuch mit Bakteriophagen stellt für einige Patienten möglicherweise eine lebensrettende Behandlung dar. Als Status quo ist er vor allem vor dem Hintergrund von Gerechtigkeitserwägungen dauerhaft nicht tragbar. Wiederholte individuelle Heilversuche könnten zudem die Grenze zur medizinischen Forschung überschreiten und stünden dann vor neuen ethischen und rechtlichen Herausforderungen [24]. Damit bleibt die Magistralrezeptur ein möglicher und ethisch wünschenswerter Zwischenschritt auf dem Weg zu einer regulären Zulassung, welche möglicherweise nur durch Studien mit angepassten Designs erreicht werden kann.

## Fazit

Dieser Beitrag konnte zeigen, dass Fragen der Aufklärung bzw. Voraussetzungen für die Möglichkeit, eine informierte Einwilligung zu geben, aus ärztlicher Sicht vor allem epistemischer Natur sind. Diese Herausforderungen können durch eine Patientenaufklärung hinreichend überwunden werden. Das Fehlen ausreichender und standardisierter Studien verhindert die Möglichkeit einer evidenzbasierten Kosten-Nutzen-Kalkulation für den Patienten. Dies ist ethisch jedoch wünschenswert, wenn Patienten in eine Therapie einwilligen sollen. Es bestehen Möglichkeiten angepasster Studiendesigns. Bestimmte Herausforderungen bleiben jedoch bestehen. Die *magistrale Herstellung* oder *Rezeptur* ist ein möglicher und ethisch abgesicherter Weg, wenn die Patienten ausreichend über die Umstände aufgeklärt wurden. Der Status quo der Phagentherapie in Deutschland begünstigt einen Medizintourismus, der ethisch nicht akzeptabel ist, weil Zugangsgerechtigkeit nicht sichergestellt werden kann. Es ist daher notwendig, die offenen medizinischen Fragen zur Phagentherapie auf ethisch vertretbare Weise zu beantworten.

## Footnotes

1 https://www.who.int/europe/de/news/item/23-11-2023-control-antibiotic-misuse-or-the-drugs-won-t-work--warn-who-experts (zugegriffen: 29.11.2024).
